# A Stepwise Approach to the Surgical Management of Hemorrhagic Choroidal Detachments

**DOI:** 10.1177/2474126421992024

**Published:** 2021-03-02

**Authors:** Shanna C. Yeung, Ryan H. Mason, Samuel A. Minaker, Alaa AlAli, Peter J. Kertes, Peng Yan

**Affiliations:** 1 Faculty of Medicine, University of British Columbia, Vancouver, BC, Canada; 2 Department of Ophthalmology and Vision Sciences, Toronto Western Hospital, Toronto, ON, Canada; 3 Kensington Vision and Research Center, Toronto, ON, Canada; 4 Department of Ophthalmology and Vision Sciences, Sunnybrook Health Sciences Centre, Toronto, ON, Canada

**Keywords:** hemorrhagic choroidal detachment, sclerotomy, surgical technique, transconjunctival, trocar, choroidal effusion

## Abstract

**Purpose:**

This work describes a stepwise surgical approach to draining choroidal detachments and 2 cases for which this approach was used.

**Methods:**

The first step involves insertion of an anterior chamber maintainer and a nonvalved 23- or 25-gauge trocar cannula at the highest peak of hemorrhagic choroidal detachment (as determined using B-scan ultrasonography), 6 to 8 mm from and angled 20° to 30° toward the limbus. The second step involves removal of the trocar to expose the sclerotomy. Alternatively, the second step can be insertion of a second trocar. The third step involves the creation of a small focal peritomy around the preexisting sclerotomy and enlargement of the preexisting sclerotomy into a radial sclerotomy. Progression between steps only occurs if prior steps did not provide adequate drainage.

**Results:**

Two cases of appositional hemorrhagic choroidal detachments in hypotonic eyes were successfully resolved by this stepwise approach. In case 1, a choroidal detachment developed after a corneal ulcer perforation. The hemorrhagic choroidal detachment in case 1 was resolved with steps 1 and 2, and an unnecessary scleral cutdown was avoided. In case 2, a choroidal detachment developed after a trabeculectomy. The detachment in case 2 required progression to step 3, extension of the trocar insertion site into a radial sclerotomy.

**Conclusions:**

This stepwise approach should be considered to reduce excessive manipulation of the globe and conjunctiva in hemorrhagic and serous choroidal detachments that warrant surgical intervention.

## Introduction

Hemorrhagic choroidal detachments are a rare but serious complication of trauma or intraocular surgery. While most cases resolve spontaneously or with medical management, surgical drainage is generally indicated when there is an appositional hemorrhagic choroidal detachment, which can lead to retinal adhesions; persistent decreased visual acuity; a flat anterior chamber, which can lead to keratolenticular adhesion, pupil block, and peripheral anterior synechiae; or persistent or progressing choroidal detachment despite conservative therapy.^1-3^ The established drainage method is the scleral cutdown approach, which involves a radial or circumferential incision through the sclera over the region of most prominent detachment.^3^ The scleral cutdown approach is effective but invasive, involving large conjunctival cutdown, manipulation of the rectus muscle, and elevated risk of hypotony, perforation, or retinal damage.^4^ A transconjunctival approach involving a trocar system is a much more delicate and less invasive alternative.^2,4^

This article describes a stepwise surgical approach to draining choroidal detachments that begins with the transconjunctival approach, which can easily progress to a scleral cutdown approach if necessary. Two cases are provided to illustrate key features of this approach. In both cases, an appositional hemorrhagic choroidal detachment developed a hypotonic eye and was resolved with this stepwise approach.

## Methods

### The Technique

The benefits of the transconjunctival approach to choroidal drainage include small-gauge sclerotomy that is less invasive, minimal conjunctival and rectus muscle manipulation, and controlled and directly visualized drainage. Table 1 and Supplemental Video 1 detail the steps of this approach. After each major step, fluid drainage levels should be reassessed to determine whether drainage is sufficient or progression to the next step is warranted.

**Table 1.** A Step-By-Step Description of the Stepwise Approach to the Surgical Management of Hemorrhagic Choroidal Detachment.

Step	Technique	Clinical notes
1	Injection of tissue plasminogen activator (if needed)	Consider administering 12.5 to 50 µg of tissue plasminogen activator 15 min before surgery if coagulated blood impedes drainage from the suprachoroidal space
	Anterior chamber maintainer	Inject a dispersive viscoelastic device or infusion of balanced salt solution into the anterior chamber Use if the cannula placement in the vitreous cavity is uncertain or poorly visualized
	Trocar cannula insertion	To access the suprachoroidal space, insert a 23- or 25-gauge nonvalved trocar cannula at the highest peak of hemorrhagic choroidal detachment (as determined using B-scan ultrasonography), 6–8 mm from the limbus and angled at 20° or nearly parallel to the limbus (Figures 1 and 2) Use nonvalved cannulas or the vitrector to vitrectomize a valved trocar
	Increase infusion pressure	Increase the infusion pressure, either through the anterior chamber maintainer or infusion cannula placed into the vitreous cavity to promote faster outflow of the choroidal fluid
	Application of external pressure	Perform a rolling motion with cotton-tip applicators to milk the suprachoroidal fluid to the sclerotomy site and promote drainage (Figure 3)
2	Connection to active aspiration	Insert a 26-gauge guarded needle connected to active aspiration to enhance drainage
	Second trocar cannula insertion	If fluid outflow is inadequate, insert a second 23- or 25-gauge trocar cannula into a different quadrant using the same method used to insert the first trocar cannula Use B-scan to determine areas of high choroidal detachment
	Cannula removal	If fluid outflow is inadequate, remove the suprachoroidal trocar cannula to expose the sclerotomy, and keep infusion pressure high to optimize drainage
3	Extending the sclerotomy	If fluid outflow remains inadequate, use a beaver blade to extend the preexisting sclerotomy (instead of scleral cutdown) into a 2- to 3-mm radial sclerotomy (Figure 4) Spontaneous drainage of suprachoroidal fluid or hemorrhage should now occur but can be further encouraged with placing a cyclodialysis spatula in the incision, gently pressing cotton-tip applicators near the incision opening, or rolling cotton-tip applicators to milk suprachoroidal fluid to the sclerotomy site

#### Presurgical Planning

**Figure 1.**
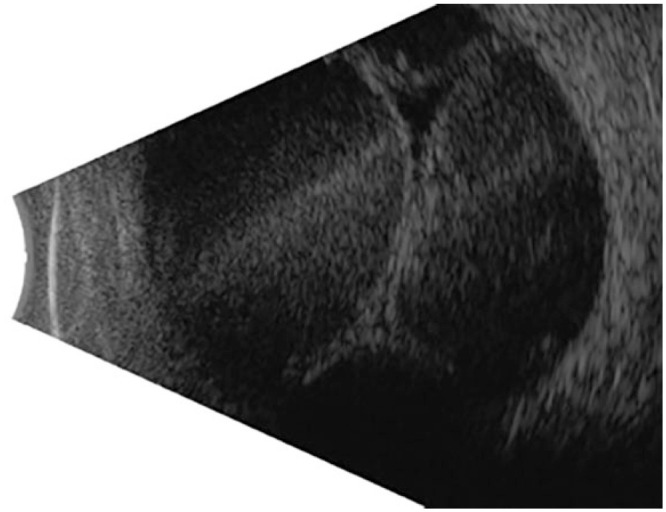
Preoperative B-scan showing appositional hemorrhagic choroidal detachment in the left eye of case 2.

Surgical drainage is ideally performed 7 to 14 days after detachment onset when the suprachoroidal hemorrhage has likely liquefied.^5^ Ultrasound biomicroscopy can be used to clinically evaluate the condition. Reduced reflectivity on A-scan ultrasonography and a more echolucent pattern with fine diffuse opacities on B-scan ultrasonography can suggest that a hemorrhage has liquefied.^6^ A preoperative B-scan helps identify the area of peak choroidal detachment that will be the target location for drainage (Figure 1).

#### Step 1

**Figure 2.**
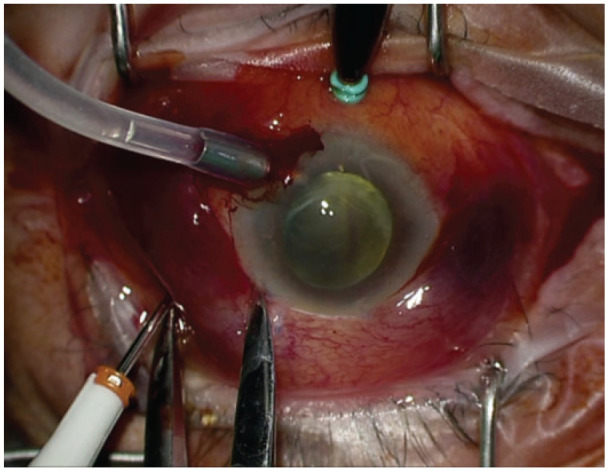
A 23-gauge valved trocar was inserted at the highest peak of hemorrhagic choroidal detachment as determined by B-scan ultrasonography, 6 to 8 mm from the limbus and angled 20° or nearly parallel to the limbus. The vitrector can be used to convert a valved trocar into a nonvalved one. Image from case 2.

**Figure 3.**
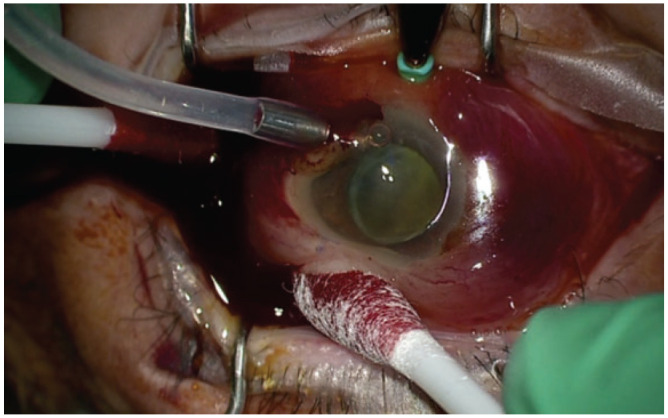
A rolling motion can be performed with cotton-tip applicators to milk the suprachoroidal fluid to the sclerotomy site and promote drainage. Image from case 2.

An anterior chamber maintainer should be inserted to pressurize the globe if the posterior infusion cannula placement cannot be visualized to avoid subretinal placement of the infusion cannula. A nonvalved 23- or 25-gauge trocar cannula should be inserted 6 to 8 mm posterior to the limbus at the highest peak of the choroidal detachment as determined by preoperative ultrasonography. The insertion of the trocar cannula should be aimed 20° to 30° toward the limbus to access the suprachoroidal space (Supplemental Video 1, Figure 2).^2,4^ A valved trocar can be vitrectomized to convert it into a nonvalved trocar. Liquefied hemorrhage or serous choroidal fluid often spontaneously drains after trocar insertion. Applying external pressure with cotton-tip applicators facilitates drainage (Figure 3).

If urgent surgical drainage is warranted and coagulated blood impedes drainage from the suprachoroidal space, consider administering 12.5 to 50 µg of tissue plasminogen activator (tPA) via transscleral injection or via trocar cannula into the suprachoroidal space. Patient case reports^7-9^ and rabbit experiments^10^ have both demonstrated that injecting tPA into the suprachoroidal space effectively and safely accelerates suprachoroidal hemorrhage clot liquefaction. Allow at least 15 minutes for the tPA to take effect before proceeding with the surgery.

#### Step 2

If drainage was inadequate following step 1, inserting a 26-gauge guarded needle connected to active aspiration can enhance drainage (see Supplemental Video 1). Removal of the suprachoroidal trocar cannulas to expose the sclerotomy allows for further drainage (see Supplemental Video 1). Alternatively, a second trocar can be inserted into a second quadrant (in a similar approach to step 1) to target the second highest peak of choroidal detachment as determined by B-scan.

#### Step 3

**Figure 4.**
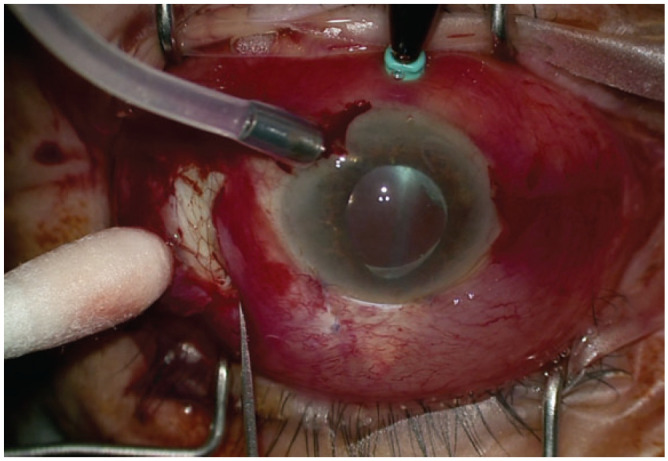
If fluid outflow remains inadequate, use a beaver blade to extend the preexisting sclerotomy (instead of scleral cutdown) into a 2- to 3-mm radial sclerotomy. Image from case 2.

If drainage is inadequate following step 2, create a small focal peritomy around the preexisting sclerotomy and enlarge the preexisting sclerotomy into a 2- to 3-mm radial sclerotomy (see Supplemental Video 1, Figure 4). Extending a preexisting sclerotomy is safer and more controlled than starting with a large radial cutdown, and it may be a safer technique when the globe is soft. Suprachoroidal fluid or hemorrhage should spontaneously drain after sclerotomy extension.

## Results

### Cases

#### Case 1

An 86-year-old man developed corneal perforation and hypotony secondary to a corneal ulcer in his right eye. The corneal ulcer was initially managed with topical antibiotics and cycloplegics, but the patient developed intractable ocular pain after 10 days. On presentation, clinical examination showed a flat anterior chamber and a full-thickness corneal perforation plugged by an incarcerated iris. The patient presented with light perception visual acuity (VA) in the affected eye. B-scan ultrasonography showed an appositional hemorrhagic choroidal detachment. Closure of the corneal perforation using 10-0 nylon suture was attempted but was only partially achieved because of the nature of the button-hole perforation and corneal melt. A large scleral cutdown was avoided because of the risk of exacerbating hemorrhage and hypotony in this already hypotonic eye.

The surgeon inserted an anterior chamber maintainer to maintain intraocular pressure (IOP), although there was some leakage through the incomplete corneal wound closure, and placed a nonvalved trocar 6 mm from the limbus in the temporal quadrant at the highest peak of choroidal detachment (as determined by preoperative B-scan). Although spontaneous suprachoroidal drainage was achieved through the trocar cannula by increased IOP, the presence of incomplete closure of corneal perforation warranted a larger drainage site. The trocar cannula was removed through the sclerotomy and the conjunctiva was opened to expose the sclerotomy. Drainage of the suprachoroidal hemorrhage was achieved and the red reflex then returned, indicating adequate resolution of the hemorrhage. Step 3, extension of the sclerotomy into a radial sclerotomy, was not needed. The corneal perforation was closed with cyanoacrylate glue and the patient was referred to a corneal specialist for a corneal transplant.

#### Case 2

**Figure 5.**
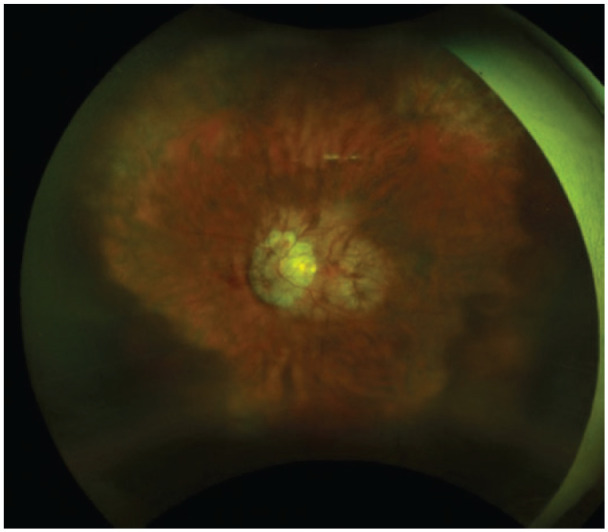
Color fundus photography at 2 months post operation showing resolution of hemorrhagic choroidal detachment. Image from case 2.

**Figure 6.**
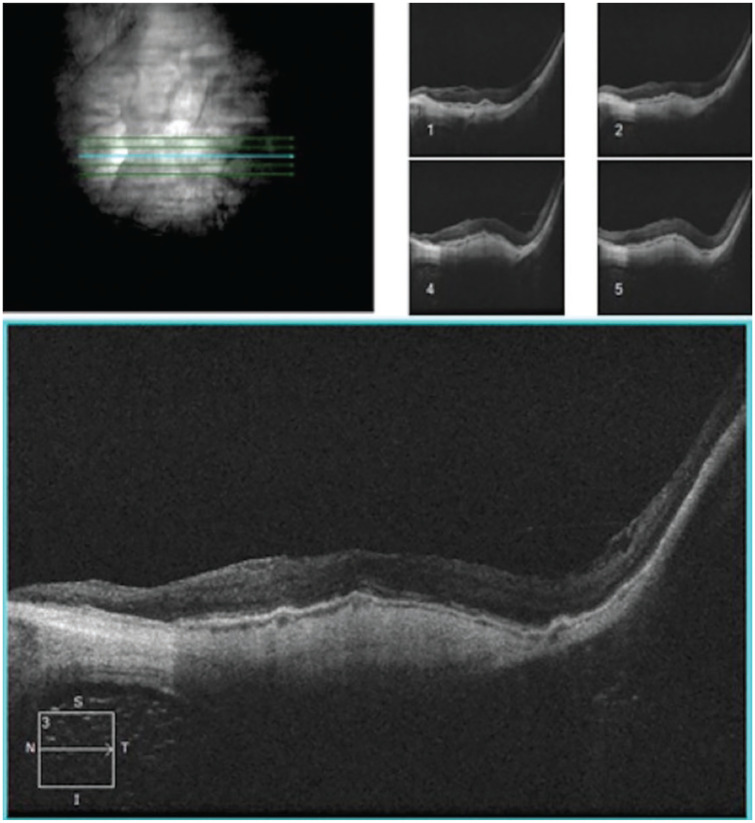
Optical coherence tomography at 2 months post operation showing resolution of hemorrhagic choroidal detachment. Image from case 2.

An 88-year-old with monocular vision secondary to advanced glaucoma with previous shunt surgery in his right eye, bilateral pathological myopia, and posterior staphyloma had a baseline VA of counting fingers in his right eye and 20/40 in his left eye. Following trabeculectomy in the left eye, he developed an appositional hemorrhagic choroidal detachment. The patient presented with counting fingers VA and an IOP of 6 mm Hg in the left eye despite medical management. The persistence of an appositional hemorrhagic choroidal detachment and pain in a patient with monocular vision. The patient was treated with all 3 steps of the aforementioned stepwise choroidal drainage approach, including extension of the sclerotomy into a radial sclerotomy to achieve complete resolution of the choroidal detachment. By postoperative week 6 the patient’s vision was 20/30. Imaging at 2 months post operation showed complete resolution of the hemorrhagic choroidal detachment (Figures 5 and 6).

## Conclusions

This article describes a stepwise approach to managing a hemorrhagic choroidal detachment that progresses from the least invasive trocar cannula insertion to extension of sclerotomy into a radial sclerotomy if adequate drainage is not achieved. This approach minimizes incision size, which reduces tissue disruption and damage compared with the traditional scleral cutdown approach. We recommend this approach in all patients with hemorrhagic choroidal detachments to reduce unnecessary risk of exacerbating hemorrhage and/or hypotony. Future studies are needed to investigate the efficacy and risk profile of this stepwise approach as compared with the conventional scleral cutdown approach.
